# The impact of physical activity and dietary behavior on depression in college students: a study on mediation effects and network analysis

**DOI:** 10.3389/fpubh.2025.1683468

**Published:** 2025-10-08

**Authors:** Wen Zhang, Wenying Huang, Chang Hu, Yuqing Yuan, Xinyi Chen

**Affiliations:** School of Physical Education, Jiangxi Normal University, Nanchang, Jiangxi, China

**Keywords:** physical activity, dietary behavior, depression, college students, mediation analysis, network analysis

## Abstract

**Objectives:**

This study investigates the relationships between physical activity, dietary behavior, and depressive symptoms among college students, focusing on the mediating role of dietary behavior and interconnections revealed by network analysis.

**Methods:**

We utilized convenience sampling to recruit 2,487 college students from five universities in Jiangxi Province, China. Data were collected through an online questionnaire. Physical activity levels were gauged through a brief inquiry, dietary behavior was evaluated using the College Students’ Dietary Health Literacy Questionnaire, and depressive symptoms were quantified using a widely recognised scale from the Centre for Epidemiological Studies (CES-D). Descriptive statistical analysis was conducted using SPSS 26.0, mediation analysis using PROCESS version 3.5, and network analysis using the R programming language.

**Results:**

A significant negative correlation was found between physical activity and depression (*β* = −0.15, *p* < 0.001), with a comparable pattern observed for dietary behavior (*β* = −0.49, *p* < 0.001). Dietary behavior served as a partial mediator in linking physical activity to depression, explaining 65% of the overall impact (*β* = 0.28, 95% CI = [0.31, 0.25]). Network analysis results indicated that three nodes, Y6, Y10, and Y15, were centrally positioned within the network. The bridge expected influence showed that physical activity was strongly associated with dietary behavior and depression clusters.

**Conclusion:**

The findings highlight significant interconnections between physical activity, dietary behavior, and depressive symptoms. The partial mediating effect of dietary behavior underscores its importance in the relationship between physical activity and depressive symptoms. These results suggest that interventions targeting both physical activity and dietary behavior could positively impact depressive symptoms in college students, offering valuable insights for preventing and managing depression in this population.

## 1 Introduction

In the face of intensified social competition and a faster pace of life, the psychological well-being of university students is receiving increasing attention (1). Navigating the critical transition from adolescence to adulthood, college students encounter multifaceted challenges, including academic pressure, interpersonal difficulties, financial hardships, career uncertainties, and personal psychological factors, rendering them highly susceptible to depression (2, 3). Relevant data reveals that the prevalence of depressive symptoms among Chinese university students rose by 6.04 percentage points between 2000 and 2017 (4). A meta-analysis further demonstrated a 34.7% prevalence rate of depressive symptoms among Chinese university students (5, 6). These statistics highlight the widespread and severe nature of depression among this demographic. Depression, as a common psychological disorder, not only affects an individual’s mental wellbeing but also has long-term adverse effects on academic performance, social interactions, and physical health (7, 8). Researches indicate that the persistent presence of depressive moods may lead to a decline in academic performance, deterioration of social skills, and worsening of physical health (9, 10). Moreover, depression can further precipitate comorbid psychological conditions, including anxiety disorders, self-harm behaviors, and suicidal tendencies. These manifestations substantially burden individuals, families, and society (11, 12). Consequently, a comprehensive investigation into the aetiology of depression among university students and the development of evidence-based interventions carries significant practical implications.

Maintaining psychological wellbeing is well established to be substantially influenced by lifestyle patterns comprising regular physical exercise and balanced nutritional practices (13–15). Physical activity promotes physiological fitness and facilitates mood regulation and alleviation of depressive and anxiety symptoms through neurochemical modulation, particularly via endorphin and serotonin pathways (16–18). Empirical studies confirm that consistent physical activity substantially attenuates depression risk while enhancing holistic quality-of-life metrics (19). Moreover, dietary patterns exert significant modulatory influences on psychological wellbeing (20, 21). Thus, this investigation employs network modeling and mediation analysis to thoroughly investigate the underlying relationships between physical activity, dietary patterns, and depressive symptoms among university students. The findings will provide a solid empirical basis for developing targeted mental health interventions that support students’ holistic development and long-term psychosocial functioning.

## 2 Literature review and hypotheses development

### 2.1 Physical activity and depression

Physical activity (PA), which is operationalised as all bodily locomotion generated by skeletal musculature requiring energy expenditure (22), constitutes not only a cornerstone of physiological health but also an essential component of emotional regulation through neurocognitive mechanisms (23, 24). It serves as an effective means of stress relief, helping individuals to break free from the pressures of daily life (25–28). Additionally, Physical activity augments self-efficacy and self-esteem, representing core psychological constructs essential for sustaining emotional regulation through neurocognitive reward pathways (29–32). Empirical syntheses confirm physical activity as an evidence-based preventative against depression (33–36). His neuroprotective relationship operates through multifactorial biological pathways, wherein exercise-induced *β*-endorphin release mediates mood elevation and depressive symptom attenuation via *μ*-opioid receptor activation (37). It also increases serotonin levels, a neurotransmitter closely associated with emotional regulation (38–40). Additionally, physical activity may indirectly influence mood by reducing inflammatory responses and improving insulin sensitivity (41–43); both of these factors are linked to the development of depression. Numerous studies have shown that physical activity is inversely related to depression (44–46). For instance, a cohort study showed that engaging in 1,200–3,000 METs-min/wk. of physical activity per week can effectively reduce the incidence of depressive symptoms (47). Moreover, a global prospective cohort study involving 267,000 participants demonstrated that increased physical activity levels significantly reduce depression risk (48). This evidence confirms that regular physical activity enhances emotional wellbeing and helps prevent the onset of depression.

### 2.2 The mediating role of dietary behavior

The Biopsychosocial Model posits that health outcomes arise from interactions among biological, psychological, and social determinants (49). Within this multifactorial framework, physical activity not only directly benefits mental health but may also mitigate depression-related manifestations by promoting nutritionally balanced dietary patterns (14, 50, 51). This process involves multiple pathways, including regulating key neurotransmitter levels and reducing inflammatory responses (52). Biologically, physical activity modulates neuroendocrine responses, exerting pronounced regulatory effects on the hypothalamic–pituitary–adrenal (HPA) axis (53, 54). Habitual engagement in physical activity modulates the secretion of stress-related hormones, including cortisol (55), alleviating physiological responses to stress (56, 57). This physiological regulatory mechanism can significantly decrease the likelihood of emotional eating and promote healthier dietary patterns (58, 59). Studies show that people who exercise regularly usually eat more vegetables, fruits, and whole grains and cut down on processed and high-sugar foods (60, 61). Transitioning toward more nutritionally dense dietary patterns correlates with attenuated inflammatory markers. This connection is significant given that chronic low-grade inflammation has been established as a core pathological mechanism in depression pathogenesis (62). Therefore, physical activity may mitigate depression susceptibility through dietary optimisation pathways. Based on the aforementioned theories, Current evidence indicates dietary patterns exert direct neuropsychiatric effects while serving as a biologically significant mediator between physical activity and attenuated depression susceptibility.

### 2.3 A network analysis of the relationship between physical activity and depression

Previous investigations have primarily employed conventional measurement and intervention methodologies to substantiate the physical activity-depression linkage, often utilising latent variable modeling to characterise their associations (13, 34, 48, 63, 64). However, this relationship’s mechanisms and components have not been fully explored. Although path analysis has been used to elucidate the role of mediating factors such as dietary behavior, network analysis offers a more nuanced and in-depth approach, allowing for the direct examination of connections between observed variables.

Network analysis is a robust analytical framework that models intricate systems as interconnected networks constituted by nodes and edges (65). Within psychological science, nodes typically operationalise specific symptoms or behaviors, such as depressive manifestations or dietary patterns, where edges denote statistical associations between these components. This analytical framework transcends constraints inherent to conventional latent variable models, delivering a granular perspective on component interactions (66). The network architecture defined by nodes and edge configurations reveals systemic interconnectivity patterns. Furthermore, network analytics generate diverse quantitative metrics for connection assessment (67). For example, the “bridge expected influence” index can identify nodes most likely to connect different clusters within the network (68). This is particularly useful for understanding how specific behaviors or symptoms act as bridges between different aspects of the system.

University students navigating critical transitional life stages demonstrate that the physical activity-depression association may be modulated through multiple contextual variables, including academic pressures, interpersonal dynamics, and lifestyle configurations. Network analysis can provide a comprehensive framework to explore these complex interactions. Through systematic modeling of interconnections among physical activity, dietary behavior, and depressive symptomatology, we can pinpoint pivotal network components and interaction pathways that constitute viable intervention targets for enhancing psychological wellbeing. Thus, this investigation proposes synthesising network analytics with conventional mediation methodologies, enabling granular examination of the mechanistic pathways linking physical activity to depression. Specifically, it seeks to elucidate the dynamic interdependencies among physical activity, dietary behavior, and depressive manifestations within the collegiate population.

### 2.4 The present study

**Figure 1:**
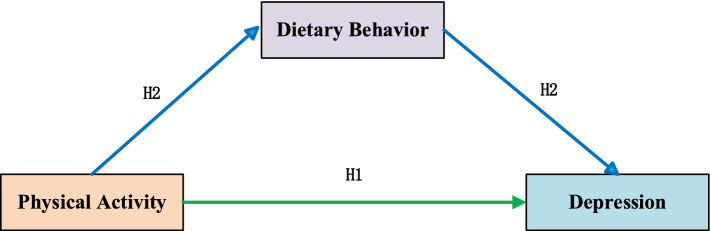
Hypothesised model diagram.

Building upon the above theoretical foundation, the present investigation postulates the following hypotheses: (1) Empirical analyses will explore the relationship between physical activity and depressive symptoms among university student populations; (2) Dietary behavior functions as a mediating pathway linking physical activity to depression outcomes in university students; (3) Network analytic approaches will intuitively clarify the interconnections between physical activity, dietary behavior, and depression. The conceptual model illustrating these hypothesised relationships appears in Figure 1.

## 3 Materials and methods

### 3.1 Participants and procedure

To establish the necessary sample size for our mediation model, we conducted a Monte Carlo power simulation targeting indirect effects, which indicated a minimum sample size of 63 participants (69). Considering the questionnaire items, we set our target sample size to 340, following the rule of 10–15 times the number of items (70). Between May and July 2025, we recruited participants via convenience sampling from five universities (three public and two private) in Jiangxi Province, China, and collected data through an online questionnaire. While convenience sampling limits the generalizability of our findings beyond similar institutional settings, it allowed us to efficiently recruit a sufficiently large sample to detect meaningful effects and ensure robust statistical power. To ensure data quality, we restricted submissions to one per IP address and excluded invalid responses (e.g., those completed too quickly or with uniform answers across all items). Out of the initial 2,746 questionnaires collected, 2,487 valid questionnaires were retained. The final sample included college students aged 18 to 30 (*M* = 20.74, SD = 2.57). Regarding gender distribution, there were 941 males (37.84%) and 1,546 females (62.16%). Regarding educational level, 2,203 participants were undergraduates or associates (88.58%), and 284 were master’s students or above (11.42%). Regarding household registration, 1,039 participants were from urban areas (41.78%), and 1,448 were from rural areas (58.22%). All participants submitted written informed consent documents, guaranteeing participant anonymity, strict data confidentiality, and unconditional withdrawal rights throughout the study. Formal authorisation for this investigation was obtained from the Institutional Review Board (IRB-JXNU-PEC-2025014), with procedures strictly adhering to the Declaration of Helsinki.

### 3.2 Measures

#### 3.2.1 Physical activity

Physical activity was assessed via a single self-report item: “Over the past 7 days, how many days did you engage in exercise or physical activity lasting at least 20 min that caused sweating or heavy breathing?” Participants reported the number of days (ranging from 0 to 7). This measurement approach has been previously validated in epidemiological research (71–73).

#### 3.2.2 Depression

Depressive symptoms were assessed using the validated Centre for Epidemiological Studies Depression Scale (CES-D), a well-established instrument introduced by Radloff in 1977 (74). This 20-item measure contains 16 negative-affect items (e.g., “I do not feel like eating; my appetite is poor.”) and 4 positive-affect items (e.g., “I feel hopeful about the future.”). Responses are recorded on a 4-point continuum, with negative-affect items scored 0 (rarely) to 3 (consistently) and positive-affect items reverse-coded. Elevated total scores correspond to greater depressive severity. Within the present sample, the CES-D exhibited outstanding internal reliability (*α* = 0.913).

#### 3.2.3 Dietary behavior

Dietary behavior was evaluated using the College Students’ Dietary Health Literacy Instrument by Wang Jiangqi et al. (75). This 13-item measure assesses three domains: Information acquisition (e.g., “I mainly learn nutrition and diet knowledge from new media.”), Information comprehension (e.g., “I understand the concept of ‘balanced diet’”), and Information application (e.g., “I’m willing to spend extra time or money on healthy meals.”). Using a 5-point Likert response format (1: Strongly disagree; 5: Strongly agree), where elevated scores reflect healthier dietary practices. Internal consistency in this cohort was excellent (*α* = 0.875).

### 3.3 Statistical analyses

Data management and descriptive statistics were processed using SPSS 26.0. Mediation pathways were analysed employing Hayes’ PROCESS macro (Model 4) using 5,000 bootstrap iterations. Mediation was confirmed when the 95% bias-corrected confidence interval excluded zero (76). Subsequently, network relationships among physical activity, dietary behaviors, and depression were modeled via the EBICglasso algorithm in R’s qgraph package (v1.9.5) (77). The network topology was constructed with variables represented as nodes and inter-variable associations encoded as edge weights, visually revealing interaction dynamics (66). Furthermore, Node influence was quantified through strength centrality metrics derived from qgraph’s centralityPlot function (78). Meanwhile, network robustness was verified by (v 1.5.0) and bootnet (v 1.5.1). We computed bridge strength indices, Edge-weight precision metrics, and Node centrality consistency measures to validate network estimation robustness. Thereby establishing analytical reproducibility (79).

## 4 Results

### 4.1 Common method biases

We implemented Harman’s single-factor test to preemptively evaluate common method variance (80). Unrotated exploratory factor analysis yielded four components exceeding Kaiser’s criterion (eigenvalues >1). The principal component accounted for 30.76% of observed variance (<40%), suggesting an absence of substantive methodological bias in the dataset.

### 4.2 Descriptive statistics and correlation analysis

Analyses revealed a significant inverse association between PA and depression (*r* = −0.431, *p* < 0.01). Depression levels negatively correlated with dietary behaviors (*r* = −0.577, *p* < 0.01). Conversely, dietary behaviors covaried with PA (*r* = 0.569, *p* < 0.01). Complete descriptive statistics are presented in Table 1.

**Table 1:** Descriptive statistics and correlation analysis (*N* = 2,487).

Variables	M ± SD	Skewness	Kurtosis	1	2	3
1. Physical activity	3.83 ± 1.94	−0.11	−1.13	1		
2. Depression	13.34 ± 9.29	0.98	2.87	−0.431^**^	1	
3. Dietary behavior	39.83 ± 11.11	0.01	−1.12	0.569^**^	−0.577^**^	1

** *p* < 0.01.

### 4.3 The mediation analyses

**Figure 2:**
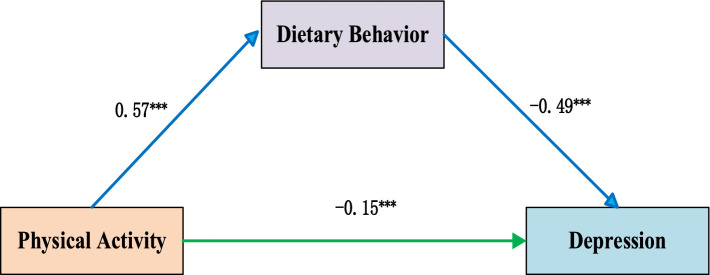
Mediation model. ^***^p < 0.001.

Our multicollinearity diagnostic revealed a maximum VIF among all variables to be only 1.479. This value falls substantially below the conventional cutoff of 10, confirming that multicollinearity posed no substantial threat to our analytical model (81). Following variable standardisation, we employed PROCESS Model 4 to examine the mediating role of dietary behavior. Analyses showed a statistically significant inverse association between physical activity and depression (*β* = −0.15, *p* < 0.001). Similarly, dietary behaviors demonstrated a robust inverse association with depression (*β* = −0.49, *p* < 0.001). Mediation analyses identified dietary behavior as a partial mediator in the physical activity–depression association [indirect effect *β* = −0.28, 95% CI (−0.31, −0.25)]. This mediated pathway explained 65% of physical activity’s total effect on depressive symptoms. However, given that our study employed a cross-sectional design, we cannot ascertain the temporal sequence of these associations and therefore cannot draw causal conclusions. A visual representation is provided in Figure 2.

### 4.4 Network analysis

**Figure 3:**
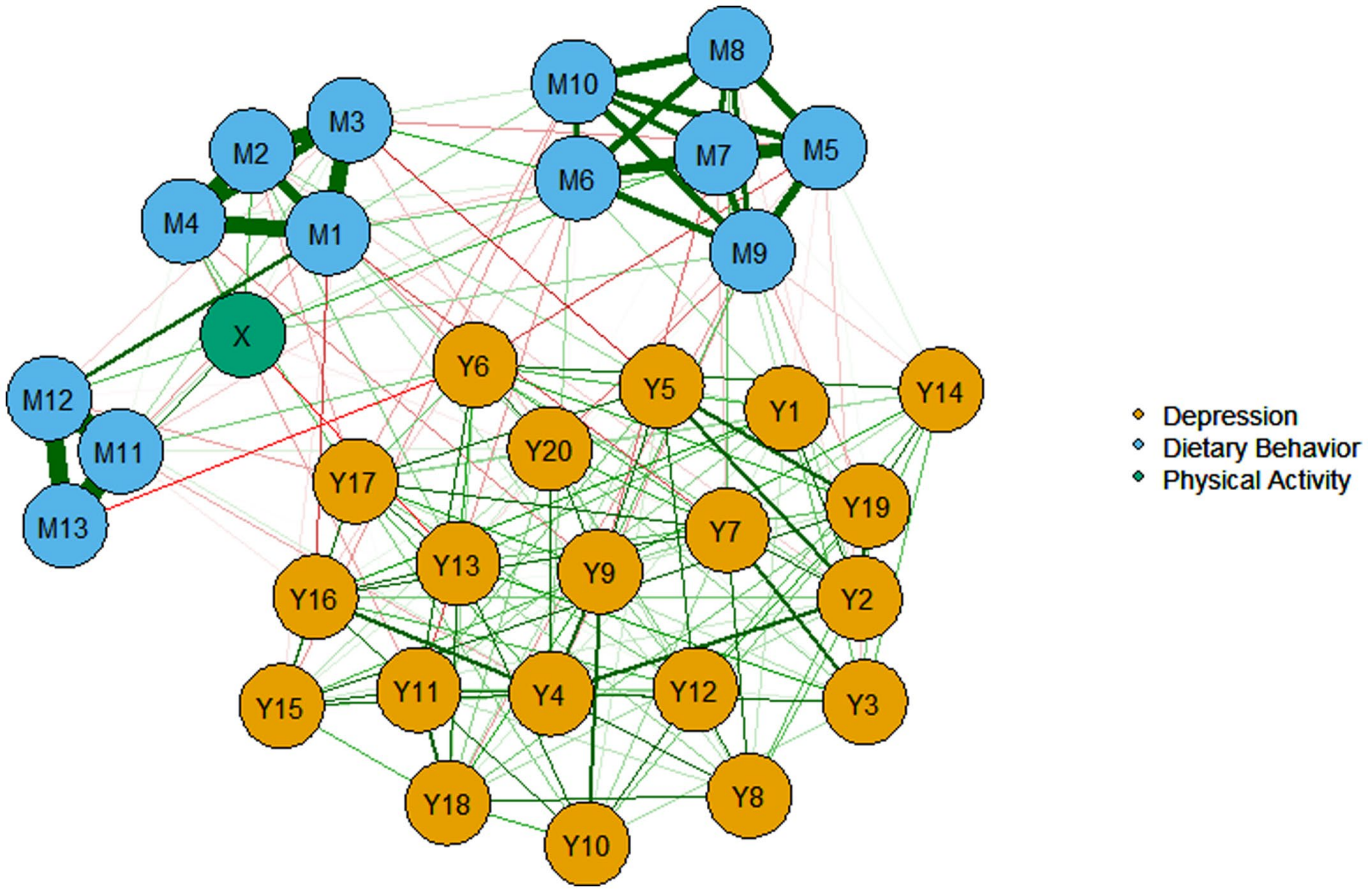
EBICglasso model based on the item-level network analysis.

Figure 3 delineates the structural relationships through which PA impacts depression. The network comprises 34 nodes and 337 non-zero edges (60%). Notably, the depression and dietary behavior networks formed relatively concentrated clusters, with closely connected internal components. The abbreviations for each variable are detailed in Appendix Table 1.

**Figure 4:**
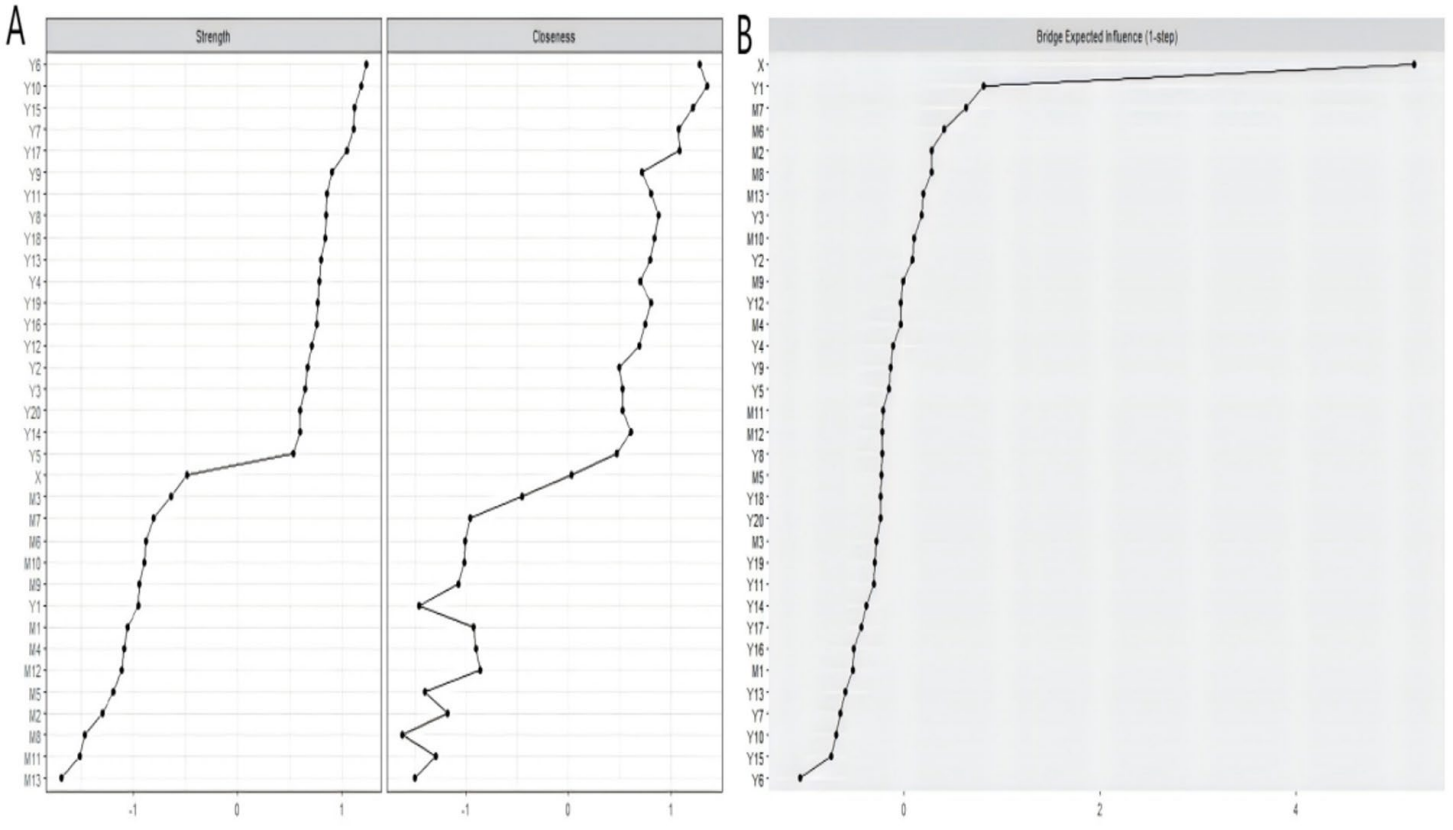
Network analysis centrality measures **(A)** and bridge expected influence plot **(B)**.

Drawing on established network methodologies (82), strength and closeness centrality metrics were selected to determine nodal influence. Figure 4A demonstrates that nodes Y6 (“I get upset over little things.”), Y10 (“My sleep is restless.”), and Y15 (“I feel that people dislike me.”) exhibit significantly elevated centrality metrics compared to other nodes, establishing these as the network’s core components. Bridge expected influence results (Figure 4B) reveal that X (“physical activity”) is strongly associated with inter-cluster connectivity between dietary behavior and depression clusters. Subsequently, Y1 (“I do not feel like eating; my appetite is poor.”) is substantially associated with bridging physical activity and dietary behavior clusters. Network stability (CS-coefficient > 0.25) confirms the reliability of bridge centrality estimates for all nodes. The relevant stability indicators are in Figures 1–3 in the Appendix.

## 5 Discussion

This research synthesised mediation modeling and psychometric network analysis to elucidate mechanistic pathways linking physical activity with depression. Analyses established physical activity as a significant antecedent of depressive symptomatology, while dietary patterns partially mediated this association. Furthermore, Psychometric network modeling determined that symptom node Y6 (“I get upset over little things.”), Y10 (“My sleep is restless.”), and Y15 (“I feel that people dislike me.”) were centrally positioned within the network. The bridge expected influence analysis showed that physical activity had a strong connection between the clusters of dietary behavior and depression.

This study demonstrated that physical activity can effectively mitigate depressive symptoms, a finding aligned with prior research (83–85). Empirical evidence has confirmed that physical activity is an effective non-pharmacological intervention for the prevention of depression (86). A meta-analysis by Schuch et al. (48) has shown that increased levels of physical activity can significantly reduce the risk of depression. Similarly, Kandola et al. (34) have emphasized the antidepressant mechanisms of physical activity through neurobiological pathways, such as the modulation of endorphins and serotonin. These studies once again confirm that physical activity can facilitate disengagement from stressors and enhance stress-coping abilities by improving overall health status, thereby achieving a mood-enhancing effect (87). In summary, physical activity is a universal protective factor against depression.

Mediation analyses substantiated dietary behavior’s role as a partial intermediary pathway between physical activity and depression, a finding that is consistent with previous research. A meta-analysis by Liang et al. (14) has shown that the combination of physical activity and dietary quality is associated with a reduced risk of depressive symptoms. Similarly, studies on Western populations have also found that healthy dietary patterns, such as the Mediterranean diet, are linked to a lower risk of depression (88). These findings further confirm the consistent relationship between physical activity, dietary behavior, and depression across different cultural contexts. Grounded in the Theory of Planned Behavior, attitudinal dispositions, perceived social norms, and behavioral control perceptions jointly govern individuals’ intentional frameworks and enacted behaviors (89). For college students, the campus environment largely shapes their dietary behaviors, while they often find it difficult to effectively intervene in key aspects such as food processing (90, 91). Therefore, understanding the healthiness of the diet from the source is key for college students to improve their dietary health level. Individuals exhibiting higher physical activity levels typically demonstrate greater health consciousness and prioritise wellness, patterns that manifest through exercise adherence and informed dietary decisions (92). The Health Belief Model further underscores how crucial it is for individuals to perceive health threats and recognize the benefits of health behaviors in shaping their behavioral choices (93). Increased physical activity may lead individuals to more profoundly recognize the benefits of healthy eating in preventing psychological problems such as depression, thereby prompting them to adopt healthier dietary behaviors. These healthy dietary behaviors provide necessary nutritional support, maintain normal brain function, and improve emotional regulation, ultimately positively impacting depressive symptoms (88, 94). Consequently, physical activity indirectly mitigates depressive risk among university populations by fostering health consciousness, strengthening behavioral intentions, and optimizing dietary patterns.

The network analysis results indicated that specific psychological and behavioral factors are central to understanding the interplay between PA, dietary behavior, and depressive symptomatology in collegiate populations. Specifically, the nodes Y6 (“I get upset over little things.”), Y10 (“My sleep is restless.”), and Y15 (“I feel that people dislike me.”) show higher strength and closeness, signifying greater inter-nodal connectivity and potential catalytic influence on system-wide mental health transitions. Further analysis of bridge expected influence suggests that X (“physical activity”) and Y1 (“I do not feel like eating; my appetite is poor.”) may serve to connect different networks within the network.

Specifically, Y6, which refers to frequently getting upset over little things, is associated with difficulties in emotion management and underscores the importance of emotional regulation in maintaining mental health. Y10 highlights the impact of sleep problems on emotions and cognitive functions. Sleep issues are associated with interference in normal circadian rhythms and neurotransmitter balance, affecting individuals’ emotional responses and daily functioning. Y15 suggests that social relationships and self-perception may be associated with the formation of depressive moods. Chinese students, who are often part of collectivist cultures, may exhibit different patterns of emotional regulation, sleep quality, and social relationships compared to Western students. For example, collectivist cultures, such as those in China, may place greater emphasis on social harmony and group cohesion, which could influence the formation and impact of depressive moods (95). In contrast, individualistic cultures, such as those in Western countries, may place more emphasis on personal autonomy and self-reliance, which could affect how individuals perceive and cope with stress and depression.

The bridge expected influence indicates that physical activity and dietary behavior are associated with mental health through multiple pathways. Physical activity, as a bridge node, is associated with improved mood, enhanced self-efficacy, and better sleep quality. The clinical manifestation Y1 (“I do not feel like eating; my appetite is poor.”) potentially reflects integrated biopsychosocial health. This empirically substantiates physical activity’s dual-pathway impact, with direct associations with depression-alleviating effects alongside and indirect influences on affective symptomatology through nutritional mediation, which is strongly associated with depression. This complex interplay suggests a dynamic feedback mechanism among physical activity, dietary behavior, and depression. Increased physical activity is associated with improved dietary behavior, which in turn is associated with reduced depressive symptoms, while the alleviation of depressive symptoms is associated with increased participation in physical activity and the maintenance of healthy eating.

In summary, this study has revealed the complex interplay between physical activity, dietary behavior, and depressive symptoms among college students through network analysis, highlighting the necessity to consider these dynamic relationships when formulating intervention strategies. It is recommended to implement multidimensional and integrated intervention measures, such as structured exercise programs, nutritional workshops, mental health support services, peer support programs, and comprehensive health campaigns, to promote students’ overall wellbeing. Additionally, based on the network analysis findings, it is suggested to focus on sleep hygiene education, social skills training, and emotional regulation workshops, which can effectively improve students’ mental health and quality of life. By adopting these targeted intervention measures, universities can create a supportive environment that fosters students’ physical and mental health, thereby more effectively preventing and alleviating depressive symptoms.

### 5.1 Strengths and limitations

This study has demonstrated multiple strengths in its design and implementation, laying a solid foundation for an in-depth investigation into the impact of dietary behavior on depressive symptoms among college students. A robust sample size has provided substantial statistical power to accurately uncover significant relationships between variables; validated measurement tools have ensured the reliability and validity of the study data across the board; and the application of diverse analytical methods has offered a unique and comprehensive perspective on understanding the underlying associations between these variables.

Nevertheless, there are several limitations to this study. First, the cross-sectional methodology inherently prevents causal inference. Subsequent investigations should implement prospective cohort designs to elucidate temporal sequencing and causal directionality among these constructs. Second, our sample primarily composed of college students from specific regions in China and recruited through convenience sampling, limits the generalizability of our findings due to potential selection bias and lack of representativeness. Thus, future research should incorporate a more diverse participant pool and consider using random sampling methods to enhance the generalizability of the results. Additionally, our measurement tools are reliable and valid, yet there remains scope for enhancing their comprehensiveness. Future studies should evaluate a broader range of additional variables, such as socioeconomic status, academic stress, personality, mental health history, and smoking or alcohol use, to gain a more comprehensive understanding of how these factors influence depressive symptoms. Moreover, utilizing multiple measurement tools to verify the consistency of the results and adopting a multidisciplinary approach can provide a more complete picture. By doing so, we can offer better theoretical support and practical guidance for preventing and addressing depression among college students.

## 6 Conclusion

This study has thoroughly examined the associations between physical activity, dietary behavior, and depression in college students through applying both mediation and network analyses. The study’s findings reveal that physical activity and depression are significantly inversely related, with dietary behavior functioning as a partial mediator. Moreover, the network analysis further revealed key nodes and connection pathways between physical activity and depressive symptoms, providing theoretical support and practical guidance for developing targeted mental health interventions.

## Data Availability

The original contributions presented in the study are included in the article/Supplementary material, further inquiries can be directed to the corresponding authors.
