# Supplementary material for: The impact of physical activity and dietary behavior on depression in college students: a study on mediation effects and network analysis
Source: Front Public Health. 2025 Oct 8;13:1683468. doi: 10.3389/fpubh.2025.1683468 (PMC12540118; doi:10.3389/fpubh.2025.1683468)
Supplement: Supplementary file 1 [file Supplementary_file_1.pdf]

Table1. The abbreviation for each variable selected in the item-level network

Variables	Abbreviation
**Components of Physical Activity**	
X:In the past 7 days, on how many days did you engage in at least 20 minutes of exercise or activity that caused you to sweat or breathe heavily?	
**Components of Dietary Behavior**	
M1:I am vulnerable to nutritional dietary advice on new media.	Information acquisition
M2:If I discuss food with others，I often refer to the information in the media.	
M3:I mainly learn nutrition and diet knowledge from new media.	
M4:I believe in the various dietary suggestions I read in the media.	
M5:I think the dietary guidelines are easy to understand.	Information comprehension
M6:I understand the concept of "balanced diet".	
M7:I can understand the information on food labels.	
M8:When I read about nutrition and diet， I don't need someone to help me understand it.	
M9:I found the nutritionist's statement easy to understand.	
M10:I understand the core items and standards in the dietary guidelines.	
M11;I refer to the label information on the food package when choosing food	Information application
M12:I can choose food according to my own nutritional status.	
M13;I'm willing to spend extra time or money on healthy meals.	
**Components of Depression**	
Y1:I do not feel like eating; my appetite is poor.	
Y2:I feel that even with family and friends around me, I still can't get rid of my inner distress.	
Y3:I have trouble keeping my mind on things.	
Y4:I feel that I am just as good as other people.	
Y5:I find it difficult to do anything.	
Y6:I get upset over little things.	
Y7:I feel hopeful about the future.	
Y8:I feel that my life has been a failure.	
Y9:I feel fearful.	
Y10:My sleep is restless.	
Y11:I feel happy.	
Y12:I talk less than usual.	
Y13:I feel lonely.	
Y14:I feel that people are unfriendly to me.	
Y15:I feel that people dislike me.	
Y16:I find life very interesting.	
Y17:I have cried before.	
Y18:I feel sad.I feel depressed.	
Y19:I feel depressed.	
Y20:I cannot continue my daily work.	

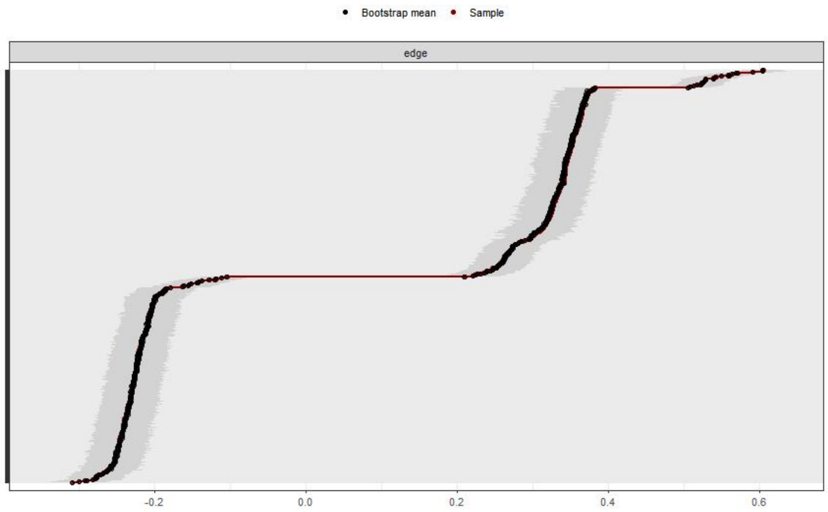

Figure 1. Accuracy of edge weights in item-level network

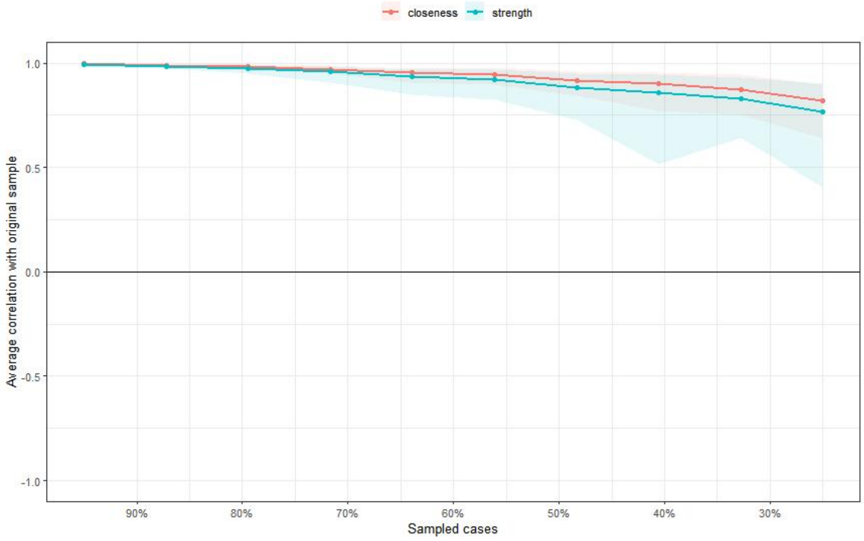

Figure 2. Stability of node closeness and strength in item-level network

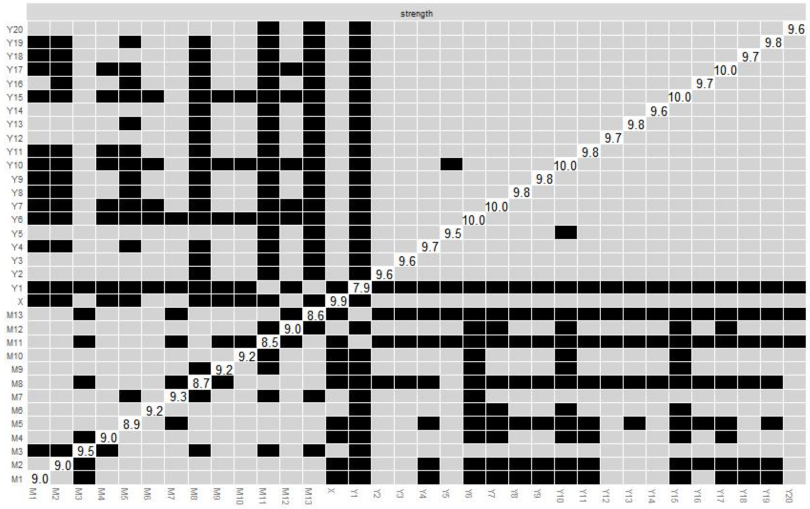

Figure 3. Bootstrapped difference test for node strength in item-level network
